# Dual Targeting of IDH2 and the Ubiquitin-Proteasome System Reveals a Functional Vulnerability in Breast Cancer Models

**DOI:** 10.3390/cancers18030368

**Published:** 2026-01-24

**Authors:** Nariman Gharari, Elisabetta Mereu, Beatrice Luciano, Bahareh Heidari, Sylvie Mader, Roberto Piva

**Affiliations:** 1 Department of Molecular Biotechnology and Health Sciences, University of Turin, 10126 Turin, Italy; nariman.gharari@unito.it (N.G.); elisabetta.mereu@unito.it (E.M.); beatrice.luciano@edu.unito.it (B.L.); 2 Institute for Research in Immunology and Cancer (IRIC), Université de Montréal, Montréal, QC H3C 3J7, Canada; bahareh.heidari@umontreal.ca (B.H.); sylvie.mader@umontreal.ca (S.M.)

**Keywords:** breast cancer, proteasome inhibitors, IDH2 inhibition, functional synergy, combinatorial treatment

## Abstract

**Background/Objectives**: Breast cancer cells rely on both mitochondrial metabolism and proteostatic mechanisms for cell fitness. The mitochondrial enzyme IDH2 supports redox balance and biosynthesis, while the ubiquitin-proteasome system (UPS) preserves protein quality. This study aimed to determine whether inhibiting IDH2 enhances sensitivity to proteasome-targeting agents across breast cancer subtypes. **Methods**: A panel of human and murine breast cancer cell lines was treated with the IDH2 inhibitor AGI-6780, alone or in combination with the proteasome inhibitor carfilzomib (CFZ) or the E1 ubiquitin-activating enzyme inhibitor TAK-243. Synergy was evaluated using Bliss scoring. Apoptosis, clonogenicity, and pathway modulation were assessed through Western blotting, colony-formation assays, and reverse-phase protein array (RPPA) profiling. **Results**: We observed that co-targeting IDH2 and the UPS produced strong synergistic cytotoxicity in multiple breast cancer models, including in triple-negative MDA-MB-231 and 4T1 cells (Bliss > 25). Combination treatments led to pronounced apoptosis, evidenced by cleaved PARP-1 and Caspase-3 cleavage, and a marked loss of clonogenic potential. RPPA analysis revealed significant alterations in key survival and stress-response pathways, including NF-κB, PI3K-p85, Src, and p38-MAPK. **Conclusions**: Inhibition of IDH2 markedly enhances the cytotoxic effects of proteasome-targeting by disrupting metabolic–proteostatic balance and promoting apoptotic cell death. These findings identify a growth-inhibitory effect that may be leveraged to improve functional dependency in breast cancer, particularly in triple-negative breast cancer, which currently lacks efficient drug treatments.

## Simple Summary

In recent years, proteasome inhibitors have shown strong activity in hematologic malignancies, but their application in solid tumors such as breast cancer remains limited and insufficiently characterized. To identify strategies that could improve their therapeutic impact, we examined whether inhibition of the mitochondrial enzyme IDH2 creates a vulnerability to proteasome blockade. Across several breast cancer models, including aggressive triple-negative subtypes, dual IDH2-proteasome inhibition induced substantially stronger apoptotic responses than either agent alone. These findings reveal a metabolic dependency that may be exploited to enhance therapeutic sensitivity to proteasome inhibitors in triple-negative breast cancer.

## 1. Introduction

Cancer cells acquire a set of biological capabilities, known as the hallmarks of cancer, that allow them to proliferate in an unchecked manner, evade cell death, adapt to hostile microenvironments, and sustain long-term survival [1,2]. Among these traits, the deregulation of cellular metabolism has emerged as a defining driver of tumor progression [3]. In this context, mitochondrial redox homeostasis represents a critical survival mechanism that enables cancer cells to tolerate oncogenic and metabolic stress. Isocitrate dehydrogenase 2 (IDH2), a mitochondrial tricarboxylic acid (TCA) cycle enzyme, catalyzes the conversion of isocitrate to α-ketoglutarate within mitochondria while contributing to NADPH production [4,5]. Mutations in IDH2 and other genes related to metabolism, such as succinate dehydrogenase (SDH) and fumarate hydratase, are found in many cancers that exhibit metabolic vulnerabilities [6,7,8,9]. While mutant IDH2 has been extensively studied in hematologic malignancies due to its production of the oncometabolite 2-hydroxyglutarate, the majority of solid tumors predominantly express the wild-type enzyme, which functions not as a direct oncogenic driver but as a metabolic safeguard supporting mitochondrial redox balance and biosynthetic capacity under oncogenic stress [10,11]. Wild-type IDH2 is highly expressed in multiple cancer types, including glioblastoma, lung cancer, and breast cancer, where it contributes to tumor growth and survival [12,13,14,15,16,17,18].

Cancer cells are highly dependent on the ubiquitin–proteasome system (UPS) to cope with the accumulation of misfolded and damaged proteins generated by oncogenic signaling and metabolic stress [19]. Pharmacological inhibition of this system using FDA-approved proteasome inhibitors (PIs), including bortezomib, carfilzomib, and ixazomib, disrupts protein homeostasis and induces apoptotic cell death [20]. While these agents have demonstrated remarkable clinical efficacy in hematologic malignancies, particularly multiple myeloma, their therapeutic impact in solid tumors has remained limited. This limited efficacy has been attributed to poor intratumoral drug penetration, intrinsic metabolic resilience, and adaptive resistance mechanisms, including enhanced mitochondrial antioxidant capacity. In addition, dose-limiting toxicities associated with currently available PIs frequently constrain therapeutic escalation, further reducing their efficacy in solid tumor settings [21]. Together, these limitations suggest that proteasome inhibition alone is insufficient to achieve durable responses in solid malignancies.

Nevertheless, the cellular stress imposed by proteasome blockade creates vulnerabilities that can be therapeutically exploited through rational combination strategies. In multiple myeloma, inhibition of the mitochondrial enzyme IDH2 using AGI-6780 compromises mitochondrial redox homeostasis and significantly sensitizes cancer cells to proteasome inhibition, highlighting a functional interplay between metabolic and proteostatic pathways [22]. In addition to targeting the proteasome directly, upstream disruption of the ubiquitin cascade—such as inhibition of the E1 ubiquitin-activating enzyme UBA1 with TAK-243—represents an alternative strategy to impair proteostasis and enhance antitumor activity [23].

To investigate the interplay between mitochondrial metabolism and proteostatic control, we employed AGI-6780, a well-characterized allosteric inhibitor of IDH2 that reduces mitochondrial NADPH production and disrupts redox homeostasis [24]. Although initially developed to target mutant IDH2, AGI-6780 has been shown to impair wild-type IDH2 enzymatic activity and is widely used as a pharmacological tool to interrogate IDH2-dependent metabolic pathways [25].

Here, we demonstrate that dual targeting of IDH2 and the ubiquitin–proteasome system—using sublethal concentrations of AGI-6780 in combination with either the proteasome inhibitor carfilzomib or the E1 ubiquitin-activating enzyme inhibitor TAK-243—significantly enhances the sensitivity of breast cancer cells to proteasome blockade in vitro. These findings support a functional interplay between mitochondrial metabolism and proteostatic stress responses and provide a rationale for combinatorial targeting strategies in breast cancer cell models.

## 2. Materials and Methods

### 2.1. Cell Culture and Reagents

Human breast cancer cell lines MDA-MB-231, SKBR3, MDA-MB-453, HCC1937, T47D, MCF7, BT474, and MDA-MB-468, as well as murine 4T1 carcinoma cells, were obtained from the American Type Culture Collection (ATCC, Manassas, VA, USA) or provided as gifts by P. Defilippi and V. Poli laboratories. Mycoplasma test was routinely monitored using PCR/qPCR-based assays. HCC1937, MCF7, 4T1, and T47D were cultured in RPMI-1640; MDA-MB-231, SKBR3, MDAMB453, BT474, and MDAMB468 were cultured in DMEM (EuroClone, Pero, Italy) supplemented with 10–20% fetal bovine serum (FBS; Sigma-Aldrich, St. Louis, MO, USA), 2 mM L-glutamine, and 100 U/mL penicillin/100 μg/mL streptomycin (Gibco, Thermo Fisher Scientific, Waltham, MA, USA). Cultures were maintained at 37 °C in a humidified incubator with 5% CO_2_. Cells were seeded at a density of 1 × 10^5^ cells/mL before drug treatment. Carfilzomib (PR-171), AGI-6780, and TAK-243 were purchased from Selleckchem GmbH (Munich, Germany).

### 2.2. Cell Viability Assay (CellTiter-Glo^®^)

Cell viability was assessed using the CellTiter-Glo^®^ Luminescent Cell Viability Assay (Promega, Madison, WI, USA). For proliferation curves, MDA-MB-231 cells were plated in 96-well white plates (5000 cells/well), and viability was measured at days 0, 2, and 4. For drug-response assays, cells were similarly seeded, allowed to adhere overnight, and treated with TAK-243, AGI-6780, carfilzomib, or DMSO for 48 or 96 h. An equal volume of CellTiter-Glo^®^ reagent was added (1:1), plates were shaken for 2 min, incubated for 10 min at RT, and luminescence was recorded using a BioTek Synergy™ 2 plate reader (Agilent Technologies, Santa Clara, CA, USA). Values were normalized to vehicle controls. All assays were performed in 3 independent biological replicates.

### 2.3. Annexin V-FITC/PI Apoptosis and Cell Cycle Analysis

Apoptosis was quantified by flow cytometry using Annexin V-FITC and Propidium Iodide (PI) staining kits (Miltenyi Biotec GmbH, Bergisch Gladbach, Germany) according to the manufacturer’s protocol. For cell-cycle analysis, cells were washed with PBS, treated with RNase A (0.14 mg/mL), and stained with PI (28.57 μg/mL). Samples were acquired on BD Accuri or BD FACS instruments, and data were analyzed using FACSDiva v8.0 (BD Biosciences, San Jose, CA, USA).

### 2.4. Colony Formation Assay

Long-term proliferative potential was assessed by colony formation assays. Cells were seeded at low density (5000 cells per 15 cm dish), allowed to adhere overnight, and treated as indicated. Plates were incubated for 14 days with medium exchanged every 3–4 days. Colonies were fixed with 4% paraformaldehyde (15 min), stained with 0.5% crystal violet (30 min), washed, and air-dried. For quantification, crystal violet was solubilized with 10% acetic acid, and absorbance was measured at 480 nm using BioTek Synergy™ 2 plate reader (Agilent Technologies). Experiments were performed in biological triplicate.

### 2.5. Western Blotting

Cells were lysed in buffer containing 20 mM Tris-HCl (pH 7.4), 150 mM NaCl, 5 mM EDTA, 1% Triton X-100, 1 mM PMSF, 10 mM NaF, 1 mM Na_3_VO_4_, and protease/phosphatase inhibitors (Roche Diagnostics GmbH, Mannheim, Germany). Lysates were incubated on ice for 30 min and centrifuged at 13,000 g for 15 min at 4 °C. Protein concentrations were quantified using the DC™ Protein Assay (Bio-Rad Laboratories, Hercules, CA, USA). After migration of equal protein amounts for each sample by SDS-PAGE and transfer to nitrocellulose membranes (GE Healthcare, Chicago, IL, USA), the membranes were blocked in 5% milk/PBST, incubated overnight at 4 °C with primary antibodies in 5% BSA/PBST, washed, and incubated with HRP-conjugated secondary antibodies for 1 h. Signals were visualized using Immobilon HRP chemiluminescent substrate (Millipore, Burlington, MA, USA). Primary antibodies PARP-1, cleaved PARP-1, caspase-3, cleaved caspase-3, IDH2, α-tubulin, and vinculin were obtained from Cell Signaling Technology (Danvers, MA, USA).

### 2.6. Quantitative RT-qPCR

Total RNA was extracted using the RNeasy Mini Kit (Qiagen GmbH, Hilden, Germany) and treated with RQ1 RNase-free DNase (Promega). cDNA synthesis was performed using iScript™ Supermix (Bio-Rad Laboratories) or SuperScript™ III Reverse Transcriptase (Invitrogen, Thermo Fisher Scientific, Waltham, MA, USA). qPCR reactions were carried out in 384-well plates using a CFX384 Real-Time PCR System (Bio-Rad Laboratories) with iQ™ SYBR Green Supermix (Bio-Rad Laboratories) under the following conditions: 95 °C for 5 min, followed by 40 cycles at 94 °C for 10 s and 60 °C for 30 s. Each reaction was run in technical triplicate. Gene expression levels were calculated using the ΔΔCt method and normalized to GAPDH. The following primer sequences were used directly in the reactions: GAPDH forward 5′-TCTTTTGCGTCGCCAGCCGAG-3′, reverse 5′-TGACCAGGCGCCCAATACGAC-3′; IDH2, forward 5′ CGGAGATGTGCAGTCAGACA-3′, reverse 5′-GCCTCAGCCTCAATCGTCTT-3′.

### 2.7. Reverse-Phase Protein Array (RPPA)

RPPA analysis was performed at the Functional Proteomics Core Facility, MD Anderson Cancer Center (Houston, TX, USA). MDA-MB-231 cells were seeded at 1 × 10^5^ cells/6 cm dish and treated with vehicle only (DMSO 0.05%, carfilzomib (10 nM), AGI-6780 (10 μM), or their combination. Cells were collected at 24 and 48 h, lysed in RPPA buffer (1% Triton X-100, 50 mM HEPES pH 7.4, 150 mM NaCl, 1.5 mM MgCl_2_, 1 mM EGTA, 100 mM NaF, 10 mM sodium pyrophosphate, 1 mM sodium orthovanadate, protease/phosphatase inhibitors). Protein concentrations were normalized to 1.5 μg/μL, mixed with 4× SDS sample buffer and β-mercaptoethanol, heated at 95 °C for 5 min, and stored at −80 °C until shipment on dry ice. Samples were processed following standard MD Anderson RPPA protocols.

### 2.8. Statistical Analysis

Statistical analyses were performed using GraphPad Prism software v9.0 (GraphPad Software LLC, San Diego, CA, USA). Two-group comparisons used an unpaired Student’s t-test; multi-group comparisons used one-way ANOVA with an appropriate post hoc comparison test. Significance was defined as *p* < 0.05. Data represent mean ± SD from at least three independent biological replicates.

## 3. Results

### 3.1. Synergistic Antitumor Effects of IDH2 and Proteasome Inhibitors Across Breast Cancer Models

**Figure 1:**
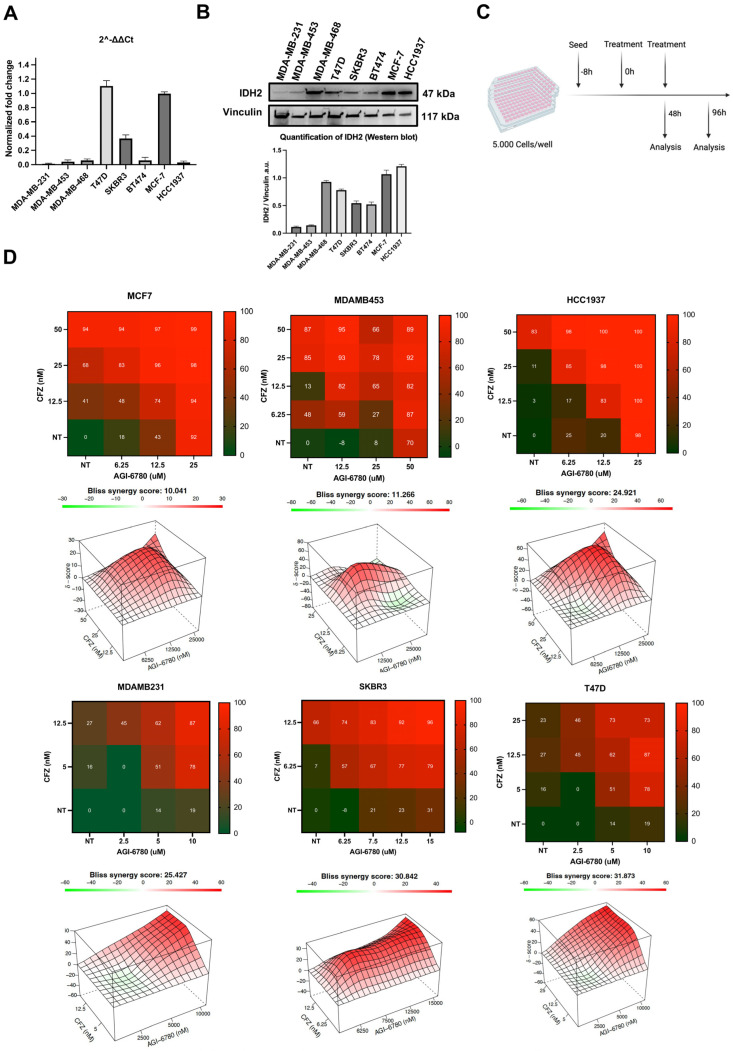
AGI-6780 Synergizes with Carfilzomib to Suppress Viability of Breast Cancer Cell Lines. (**A**) RT-qPCR analysis of IDH2 mRNA expression calculated via 2^−ΔΔCt^ and normalized to GAPDH. (**B**) Western blot analysis of IDH2 expression across the cell line panel. Vinculin was used as a loading control. Quantification of IDH2 normalized to Vinculin is shown (a.u., arbitrary units). (**C**) Experimental workflow for the combinatorial treatment of breast cancer cell lines with AGI-6780 and Carfilzomib (CFZ). Cells were seeded at 5000 cells per well and treated every 48 h over 4 d. Samples were collected for CellTiter-Glo analysis (48 h, 96 h). (**D**) CellTiter-Glo dose–response matrices after combination treatment in six breast cancer cell lines. The color intensity reflects a reduction in CellTiter-Glo values. Corresponding 3D response models were generated by Bliss synergy analysis. Data represent mean values of three independent experiments. Raw dose–response data with standard deviations for all combinations are provided in Supplementary Table S1. Synergy scores were interpreted as follows: >10 = synergistic; −10 to +10 = additive; <−10 = antagonistic.

To investigate whether targeting IDH2 can potentiate the efficacy of proteasome inhibition in breast cancer, we first assessed IDH2 expression across a panel of cell lines by RT-qPCR and Western blot analysis. Both methods revealed differential expression of IDH2 at the protein and transcript levels (Figure 1A,B). All cell lines expressed IDH2, albeit at varying levels (Figure 1A,B). ER-positive cell lines T47D and MCF7 exhibited the highest IDH2 mRNA expression and high protein levels. In contrast, the mesenchymal cell line MDA-MB-231 expressed low IDH2 mRNA and protein levels. Surprisingly, the HER2+ MDA-MB-453 and the triple-negative HCC1937 cell line had high IDH2 protein levels despite low RNA levels. We then evaluated the combination of the IDH2 inhibitor AGI-6780 and the proteasome inhibitor Carfilzomib (CFZ) across the six breast cancer cell lines. Cells were seeded and treated every 48 h with either AGI-6780, CFZ, or their combination, and were monitored for up to 96 h using CellTiter-Glo assays (Figure 1C,D). Synergy between AGI-6780 and CFZ was assessed using a 6 × 6 dose–response matrix and calculated via Bliss synergy models. All six breast cancer cell lines exhibited variable but consistent synergistic responses, with synergy scores exceeding the threshold of 10 in several combinations, indicating synergistic trends based on Bliss analysis. No correlation was observed between IDH2 mRNA or protein levels and Bliss synergy scores (RNA: Pearson r < 0.3, *p* > 0.05; WB: Pearson r < 0.1, *p* > 0.05). Indeed, cell lines expressing low (MDA-MB-231), medium (SKBR3), or high (T47D) protein levels of IDH2 had high synergy scores. As these cell lines represent different breast cancer subtypes, these results suggest sensitivity to mitochondrial perturbation in combination with proteasome stress in breast tumor cells (Figure 1D) and support a functional sensitivity of breast cancer cells to combined mitochondrial perturbation and proteasome stress.

### 3.2. Potent Synergy Between AGI-6780 and Carfilzomib in Triple-Negative Breast Cancer Cells

**Figure 2:**
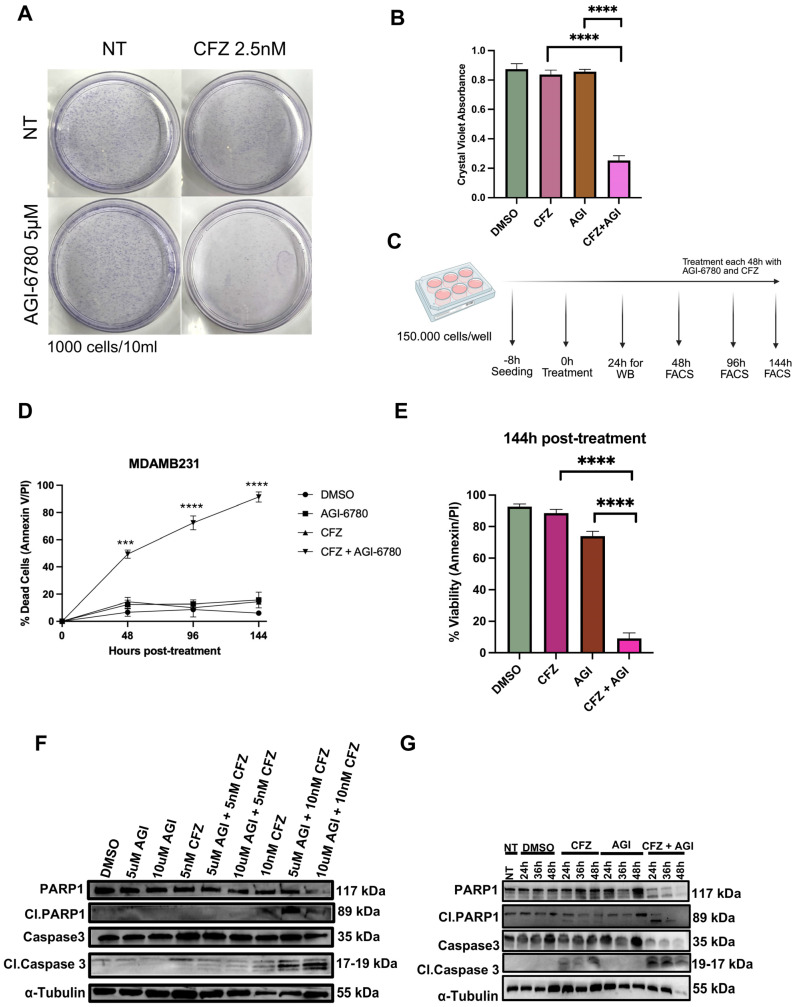
Combination of AGI-6780 and carfilzomib induces apoptosis and suppresses clonogenic survival in TNBC. (**A**,**B**) Representative images of colony formation assay. MDA-MB-231 cells were seeded at 1000 cells/well and treated with vehicle (DMSO 0.05%), AGI-6780 (5 μM), CFZ (2.5 nM), or both drugs for 10 days. Colonies were stained with crystal violet. (**C**) Experimental workflow for combinatorial treatment of breast cancer cell lines with AGI-6780 (10 μM) and Carfilzomib (10 nM). Cells were seeded at 150,000 cells per well and treated every 48 h. Samples were collected for FACS and Western blotting analyses. (**D**,**E**) Flow cytometry analysis of apoptosis. Graph showing the percentage of dead cells (Annexin V+ and/or PI+) at 144 h post-treatment with AGI-6780 (10 μM) and Carfilzomib (10 nM). Statistical analysis was performed using one-way ANOVA (*** *p* < 0.001, **** *p* < 0.0001). (**F**) Western blotting analysis of apoptotic markers. Protein lysates were collected 24 h post-treatment and analyzed for cleaved PARP-1, cleaved Caspase-3, and total Caspase-3. α-Tubulin was used as a loading control. (**G**) Western blotting analysis of apoptotic markers at three time points. Protein lysates were analyzed for cleaved PARP-1, cleaved Caspase-3, and total Caspase-3. Tubulin was used as a loading control.

We then focused on the MDA-MB-231 cell line as a model of aggressive triple-negative breast cancer to further investigate the potential synergistic cytotoxic effect of IDH2 and proteasome inhibition. To evaluate the long-term clonogenic capacity of MDA-MB-231 cells, a colony formation assay was performed. Cells were seeded at low density (1000 cells per 10 mL) and were either treated with vehicle (DMSO 0.05%), AGI-6780 (AGI, 5 µM), CFZ (2.5 nM), or their combination for 10 days. After incubation, the colonies were fixed and stained with crystal violet (Figure 2A). Colonies were fixed, stained with crystal violet, solubilized, and quantified using the colorimetric readout. Colony areas were measured and normalized to the vehicle control. Quantification of this assay (Figure 2B) revealed a significant reduction in colony-forming ability upon dual treatment with AGI and CFZ, whereas AGI or CFZ alone led to only a partial decrease. This indicates that the dual treatment results in a relative reduction in the long-term proliferative capacity of MDA-MB-231 cells under the tested conditions. Annexin V/PI flow cytometry analysis was carried out at 2, 4, or 6 days after treatment with single agents or their combination (Figure 2C). A marked increase in dead cells, either apoptotic (Annexin V+/PI−) or late apoptotic/necrotic (Annexin V+/PI+) was observed in the combination group (AGI + CFZ), as compared to vehicle, AGI, or CFZ alone (Figure 2D,E), reaching more than 80% in the combination group after 6 days (**** *p* < 0.0001) while either single agent elicited only mild effects. To examine whether the cell death observed was due to early onset apoptosis, PARP-1 and Caspase-3 cleavage analysis was performed after 24 h of treatment. The combination treatment led to strong cleavage of PARP-1 (from 117 kDa to 89 kDa) and Caspase-3 (35 kDa to 17–19 kDa), confirming apoptotic activation. These cleavages were less prominent in the AGI or CFZ groups alone, supporting a synergistic apoptotic effect of the combined treatment (Figure 2F). To evaluate the kinetics of apoptosis induction, we performed a time-course analysis in MDA-MB-231 cells treated with 10μM AGI-6780, 10nM CFZ, or their combination. The combined treatment resulted in a marked increase in cleaved PARP and cleaved Caspase 3 levels compared to either agent alone at 24 h of treatment, and continued to accumulate over time (Figure 2G). Together, these results indicate that the combination of IDH2 and proteasome inhibition synergistically induces apoptosis and impairs the clonogenic potential in triple-negative breast cancer cells. These findings highlight the therapeutic potential of targeting metabolic and proteostatic vulnerabilities in aggressive breast cancer subtypes.

### 3.3. Dual Targeting of IDH2 and the Proteasome Modulates Key Signaling Pathways and Cell Survival Mechanisms

**Figure 3:**
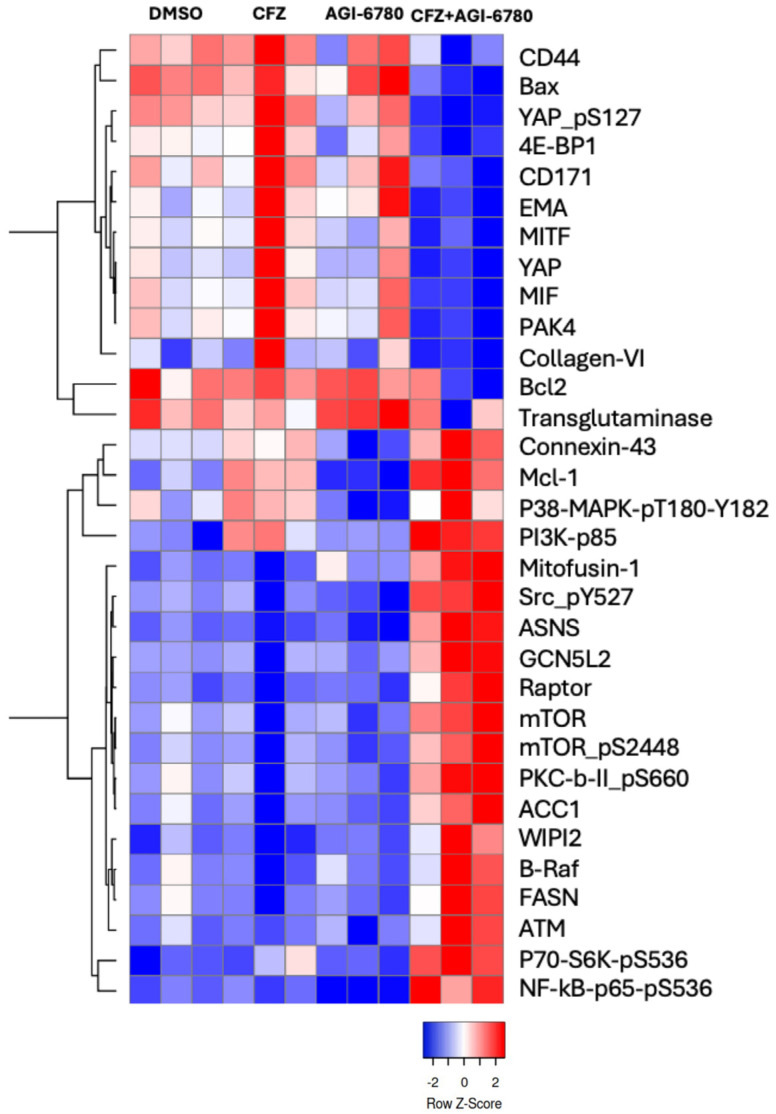
RPPA profiling and validation of selected proteins by Western blotting. Heatmap representation of differentially expressed or phosphorylated proteins identified by Reverse Phase Protein Array (RPPA) analysis in MDA-MB-231 cells treated with 10nM CFZ, 10uM AGI-6780, the combination, or vehicle. Data represent mean values from three independent biological replicates.

To investigate the molecular mechanisms underlying the synergistic effect of AGI-6780 and carfilzomib (CFZ) in inducing apoptosis of MDA-MB-231 breast cancer cells, we performed a reverse phase protein array (RPPA) analysis. The combination treatment (10 μM AGI-6780 + 10 nM CFZ for 24 h) led to significant modulation of key survival and stress-response pathways compared to the untreated condition. As indicated by the heatmap in (Figure 3), the combination of AGI-6780 and CFZ repressed several proteins overexpressed in mesenchymal TNBC cells, including YAP, L1CAM/CD171, CD44, collagen VI, as well as the pro-inflammatory cytokine MIF and RhoA regulator PAK4. Conversely, there was a pronounced upregulation of several proteins involved in cell survival and metabolic signaling pathways, including mTOR, Raptor, PKC-βII (pS660), P70-S6K (pS536), and NF-κB p65 (pS536), compared to monotherapies and the control. Notably, PI3K-p85 and NF-κB-p65 phosphorylated at Ser536 were elevated in the combination group, consistent with stress-associated signaling modulation under combinatorial treatment. In addition, p38 MAPK (T180/Y182) and Src-pY527 were also upregulated, indicating inhibitory regulation of Src signaling in response to cellular stress.

Collectively, the RPPA results indicate that the combination of IDH2 and proteasome inhibition modulates survival, stress, and apoptotic signaling pathways. This combinatorial strategy may disrupt cellular adaptability and enhance therapeutic vulnerability in triple-negative breast cancer cells.

### 3.4. Co-Targeting IDH2 and the Ubiquitin-Proteasome Pathway Induces Synergistic Growth Inhibition in MDA-MB-231 and 4T1 Cells

**Figure 4:**
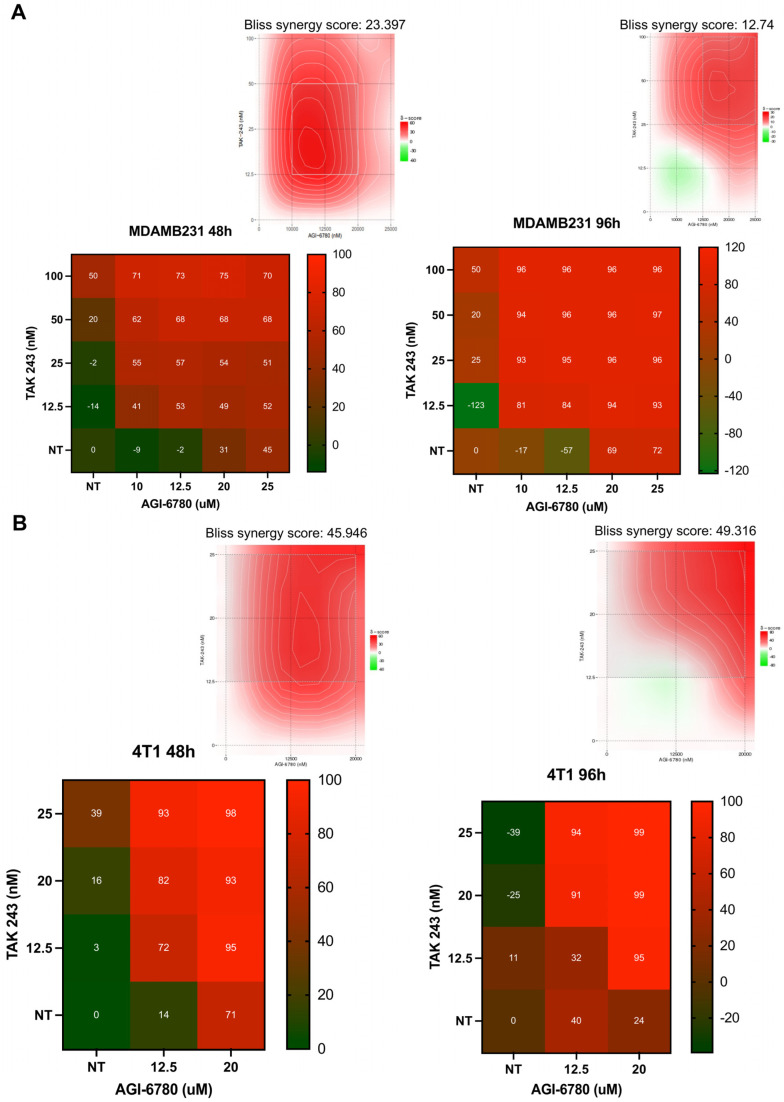
Synergistic effect of IDH2 inhibitor AGI-6780 and E1 ubiquitin-activating enzyme inhibitor TAK-243 in 4T1 and MDA-MB-231 breast cancer cells. (**A**,**B**) Heatmaps representing cell viability following single and combination treatments of AGI-6780 and TAK-243 for 48 h and 96 h in 4T1 and MDA-MB-231 cells. Synergy scores were calculated based on the Bliss independence model. Upper panels show synergy contour plots highlighting regions of synergistic interactions at different dose combinations. Red regions indicate higher synergy scores, whereas green regions indicate antagonistic effects. Raw dose–response data with standard deviations for all combinations are provided in Supplementary Table S2.

As ubiquitination targets proteins for proteasomal degradation, we also explored the potential therapeutic synergy between IDH2 inhibition by AGI-6780 and disruption of the ubiquitin system by the E1 ubiquitin-activating enzyme inhibitor TAK-243 in two TNBC breast cancer cell lines, MDA-MB-231 and 4T1 (Figure 4A,B). Cells were treated with a range of concentrations of AGI-6780 and TAK-243, both as single agents and in combination, and assessed after 48 and 96 h. Drug response matrices and synergy score heatmaps were generated using the Bliss independence model. The combination of AGI-6780 and TAK-243 consistently resulted in enhanced growth inhibition compared to single-agent treatments in both cell lines. In 4T1 cells, moderate synergistic interactions were observed after 48 h, which became more pronounced after 96 h of exposure, suggesting a time-dependent enhancement of the combinatorial effect. Similarly, in MDA-MB-231 cells, the combination treatment exhibited strong synergistic interactions, which were evident at 48 h and to a lesser degree at 96 h. The synergy contour plots further confirmed that the combination of AGI-6780 and TAK-243 led to increased efficacy.

Together, our findings suggest that targeting both IDH2 metabolic functions and the ubiquitin-proteasome system may represent a promising anti-tumor strategy in triple-negative breast cancer models.

## 4. Discussion

This study uncovered a novel and functional cellular vulnerability in triple-negative breast cancer (TNBC) by demonstrating that isocitrate dehydrogenase 2 (IDH2) inhibitor AGI-6780 synergizes with proteasome pathway disruption to enhance cytotoxic effects in vitro. Building on prior observations in hematologic malignancies [22], we extend this functional synergy framework to solid tumors, demonstrating metabolic and proteostatic co-dependencies in TNBC models.

The combination of the IDH2 inhibitor AGI-6780 with proteasome inhibitor carfilzomib (CFZ) or the E1 ubiquitin-activating enzyme inhibitor TAK-243 resulted in marked synergistic cytotoxicity in multiple breast cancer cell lines, including TNBC models MDA-MB-231 and 4T1. The synergism was quantitatively validated using Bliss models and was associated with pronounced apoptotic induction, evidenced by Annexin V/PI staining and cleavage of Caspase-3 and PARP-1. In MDA-MB-231 cells, AGI-6780 displayed stronger synergy with CFZ (Bliss ≈ 25) compared to TAK-243 (Bliss ≈ 12–23), and the effectiveness of the combination with TAK-243 decreased over time. In contrast, synergy with CFZ, which targets the terminal proteasomal bottleneck, remained more stable. In 4T1 cells, however, AGI-6780 showed robust and sustained synergy with TAK-243 (Bliss ≈ 46–49), exceeding the synergy observed in MDA-MB-231. This suggests that while the IDH2-UPS vulnerability is broadly conserved, the capacity for long-term cellular adaptation to upstream ubiquitin-blockade is model-specific. Overall, these findings underscore the sensitivity of TNBC cells to combined metabolic and proteotoxic stress.

Proteomic profiling using Reverse Phase Protein Array (RPPA) further revealed that the combined inhibition of IDH2 and proteasome activity modulates critical survival signaling pathways. Notably, upregulation of PI3K-p85, phospho-NF-κB, and phospho-Src was observed. These data suggest that induction of apoptosis is consistent with stress induced by a disruption of cellular homeostasis at the metabolic and proteostatic levels, but not genotoxic stress.

Collectively, these findings delineate vulnerabilities in TNBC that highlight functional vulnerabilities through concurrent inhibition of IDH2 and the ubiquitin/proteasome system. The data support a model in which simultaneous disruption of IDH2-sustained mitochondrial metabolism and redox balance and of the ubiquitin-proteasome pathway mediated proteostasis leads to apoptotic cell death and loss of clonogenicity. These insights offer a strong rationale for the development of combinatorial targeting approaches for targeting metabolic and proteostatic dependencies in aggressive breast cancers. This study investigated cell viability, apoptosis, and clonogenic potential induced by AGI-6780 and carfilzomib. Extending proteomic and long-term functional analyses to ER-positive and HER2-positive breast cancer subtypes will be important to expand the potential application of AGI-6780 and carfilzomib combination.

Future directions include evaluating migration, invasion, anchorage-independent growth, and in vivo tumorigenesis effects of these drug combinations, as well as assessing their tolerability and therapeutic index in xenograft models of MDA-MB-231 and of other breast cancer cell lines. Moreover, identifying biomarkers predictive of response, such as mitochondrial genes or proteasome pathway components, will be essential for patient stratification in potential clinical applications.

## 5. Conclusions

The present study highlights the functional rationale of dual IDH2 and ubiquitin-proteasome inhibition across breast cancer models, and, in particular, in TNBC. Further work is needed to clarify the underlying mechanisms of the observed synergies and advance this combinatorial approach toward clinical practice.

## Data Availability

The original contributions presented in this study are included in the article. Further inquiries can be directed to the corresponding author(s).

## Supplementary Materials

The following supporting information can be downloaded at: https://www.mdpi.com/article/10.3390/cancers18030368/s1, Table S1: Raw dose–response viability matrices for AGI-6780 and Carfilzomib combinations.; Table S2: Raw dose–response viability matrices for TAK-243 and Carfilzomib combinations.
