# Supplementary material for: Dual Targeting of IDH2 and the Ubiquitin-Proteasome System Reveals a Functional Vulnerability in Breast Cancer Models
Source: Cancers (Basel). 2026 Jan 24;18(3):368. doi: 10.3390/cancers18030368 (PMC12896526; doi:10.3390/cancers18030368)
Supplement: Supplementary file 1 [file cancers-18-00368-s001.zip › cancers-4089505-supplementary.pdf]

Supplementary Data

Dual Targeting of IDH2 and the Ubiquitin-Proteasome System Reveals a Functional Vulnerability in Breast Cancer Models

Nariman Gharari [1], Elisabetta Mereu [1], Beatrice Luciano [1], Bahareh Heidari [2], Sylvie Mader [2] and Roberto Piva [1,*]

1 Department of Molecular Biotechnology and Health Sciences, University of Turin, 10126 Turin, Italy; nariman.gharari@unito.it (N.G.); elisabetta.mereu@unito.it (E.M.); beatrice.luciano@edu.unito.it (B.L.)
2 Institute for Research in Immunology and Cancer (IRIC), Université de Montréal, Montréal, QC H3C 3J7, Canada; bahareh.heidari@umontreal.ca (B.H.); sylvie.mader@umontreal.ca (S.M.)
*Correspondence: roberto.piva@unito.it

Supplementary Table S 1. Raw dose–response viability matrices for AGI-6780 and Carfilzomib combinations. Data represent the mean percentage of cell viability ± standard deviation (SD) from three independent biological replicates. These values provide the basis for the Bliss synergy scores and heatmaps presented in Figure 1D.

MCF7 (Mean ± SD)

	AGI-6780 (µM)			
CFZ (nM)	NT	6.25	12.5	25
50	94 ± 1.6	94 ± 0.3	97 ± 1.3	99 ± 6.9
25	68 ± 2.1	83 ± 0.9	96 ± 2.3	98 ± 4.2
12.5	41 ± 2.3	48 ± 4.1	74 ± 5.1	94 ± 2.1
NT	0 ± 0.1	18 ± 2.5	43 ± 3.1	92 ± 5.1

MDA-MB-453 (Mean ± SD)

	AGI-6780 (µM)			
CFZ (nM)	NT	12.5	25	50
50	87 ± 1.0	95 ± 2.5	66 ± 3.1	89 ± 6.2
25	85 ± 0.4	93 ± 1.4	78 ± 2.3	92 ± 4.1
12.5	13 ± 3.0	82 ± 0.6	65 ± 3.2	82 ± 3.9
6.25	48 ± 2.2	59 ± 1.2	27 ± 2.5	87 ± 0.4
NT	0 ± 1.6	-8 ± 9.1	8 ± 3.8	70 ± 1.1

HCC1937 (Mean ± SD)

	AGI-6780 (μM)			
CFZ (nM)	NT	6.25	12.5	25
50	83 ± 3.2	98 ± 0.2	100 ± 3.2	100 ± 6
25	11 ± 1.3	85 ± 2.4	98 ± 5.1	100 ± 4.6
12.5	3 ± 1.8	17 ± 1.3	83 ± 3.2	100 ± 2.1
NT	0 ± 0.7	25 ± 2.1	20 ± 2.2	98 ± 0.8

MDA-MB-231 (Mean ± SD)

	AGI-6780 (μM)			
CFZ (nM)	NT	2.5	5	10
12.5	27 ± 2.1	45 ± 0.8	62 ± 4.3	87 ± 4.5
5	18 ± 1.8	0 ± 3.9	51 ± 1.7	79 ± 2.1
NT	0 ± 0.6	0 ± 1.5	14 ± 2.1	19 ± 2.7

SKBR3 (Mean ± SD)

	AGI-6780 (μM)				
CFZ (nM)	NT	6.25	7.5	12.5	15
12.5	66 ± 2.6	74 ± 2.9	83 ± 2.9	92 ± 0.6	96 ± 2.1
6.25	7 ± 1.1	57 ± 3.1	67 ± 1.6	77 ± 0.3	79 ± 1.3
NT	9 ± 3.1	-6 ± 5.7	21 ± 3.2	23 ± 1.4	31 ± 2.5

T47D (Mean ± SD)

	AGI-6780 (μM)			
CFZ (nM)	NT	2.5	5	10
25	23 ± 0.8	46 ± 1.8	73 ± 3.1	73 ± 0.6
12.5	27 ± 2.6	45 ± 1.7	62 ± 0.6	87 ± 1.8
5	16 ± 1.4	0 ± 0.8	51 ± 1.3	78 ± 2.5
NT	0 ± 0.4	0 ± 0.1	14 ± 0.3	19 ± 3.1

Supplementary Table S 2. Raw dose–response viability matrices for TAK-243 and Carfilzomib combinations. Data represent the mean percentage of cell viability ± standard deviation (SD) from three independent biological replicates. These values provide the basis for the Bliss synergy scores and heatmaps presented in Figure 4.

MDA-MB-231, 48 h

	AGI-6780 (µM)				
TAK-243 (nM)	NT	10	12.5	20	25
100	50 ± 2.1	71 ± 0.8	73 ± 1.1	75 ± 2.1	70 ± 3.9
50	20 ± 0.9	62 ± 1.8	68 ± 0.8	68 ± 5.7	68 ± 4.4
25	-2 ± 3.1	55 ± 2.6	57 ± 1.9	54 ± 4.2	51 ± 1.9
12.5	-14 ± 2.0	41 ± 0.9	53 ± 2.9	49 ± 2.7	52 ± 0.1
NT	0 ± 0.4	-9 ± 2.4	-2 ± 0.3	31 ± 3.1	45 ± 6.1

MDA-MB-231, 96 h

	AGI-6780 (µM)				
TAK-243 (nM)	NT	10	12.5	20	25
100	50 ± 0.6	96 ± 0.9	96 ± 2.0	96 ± 1.1	96 ± 6.0
50	20 ± 1.1	94 ± 0.3	96 ± 3.1	96 ± 2.8	97 ± 2.8
25	25 ± 3.1	93 ± 3.1	95 ± 0.4	96 ± 0.3	96 ± 4.2
12.5	-123 ± 11.1	81 ± 2.6	84 ± 2.7	94 ± 0.8	93 ± 2.1
NT	0 ± 0.4	-17 ± 4.7	-57 ± 5.3	69 ± 2.7	72 ± 0.5

4T1, 48 h

	AGI-6780 (µM)		
TAK-243 (nM)	NT	12.5	20
25	39 ± 3.1	93 ± 2.1	98 ± 4.6
20	16 ± 0.2	82 ± 3.4	93 ± 1.3
12.5	3 ± 0.7	72 ± 0.4	95 ± 0.2
NT	0 ± 1.2	14 ± 1.4	71 ± 2.6

4T1, 96 h

	AGI-6780 (μM)		
TAK-243 (nM)	NT	12.5	20
25	-39 ± 0.1	94 ± 1.1	99 ± 1.0
20	-25 ± 0.5	91 ± 0.5	99 ± 0.5
12.5	11 ± 0.3	32 ± 2.1	95 ± 2.1
NT	0 ± 1.7	40 ± 3.8	24 ± 3.1
